# Supplementary material for: Integrative Identification of Genetic Loci Jointly Influencing Diabetes-Related Traits and Sleep Traits of Insomnia, Sleep Duration, and Chronotypes
Source: Biomedicines. 2022 Feb 2;10(2):368. doi: 10.3390/biomedicines10020368 (PMC8962243; doi:10.3390/biomedicines10020368)
Supplement: Supplementary file 1 [file biomedicines-10-00368-s001.zip › Figure S1.pdf]

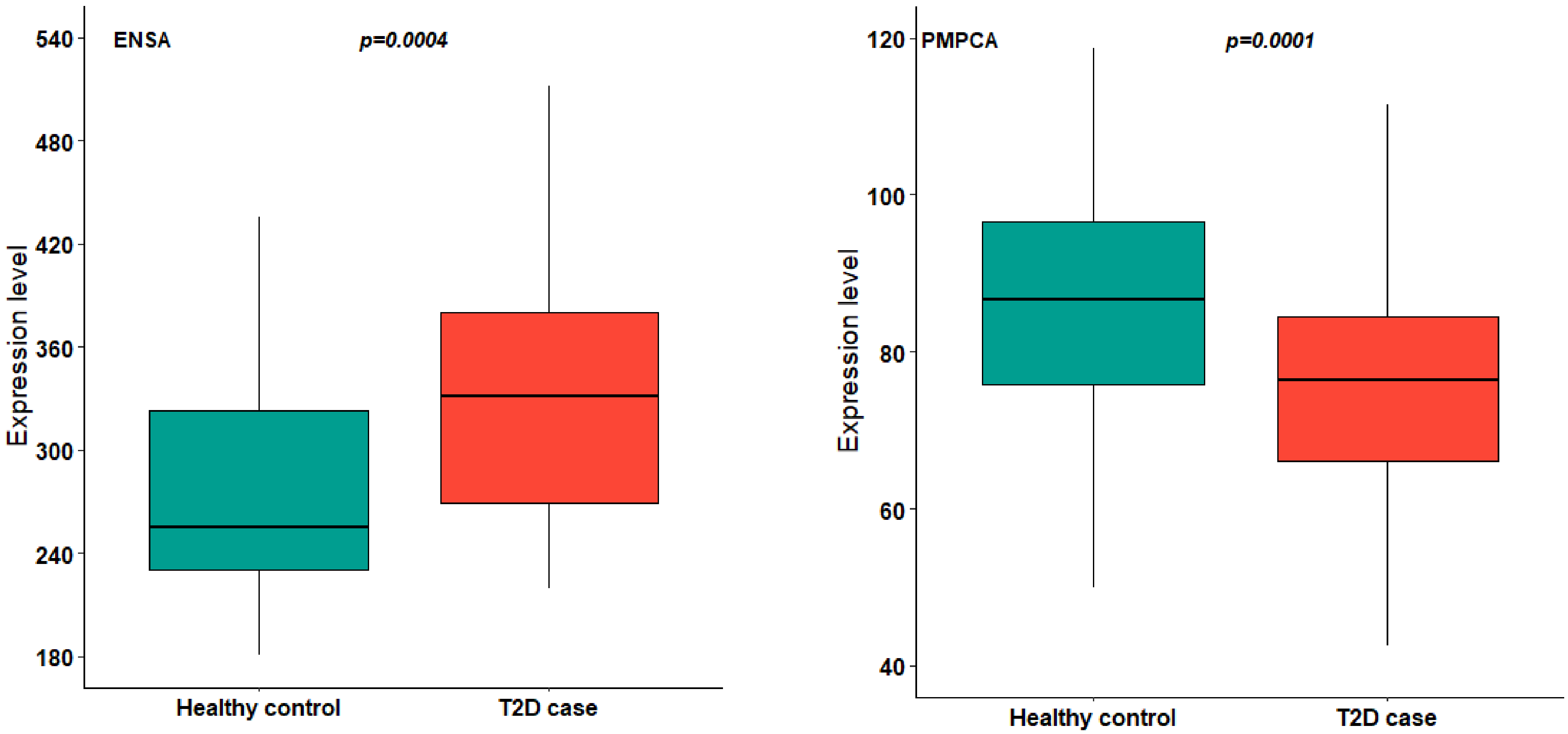

Supplementary Figure S1. Dysregulation of pleiotropic genes *ENSA* (A) and *PMPCA* (B) in type 2 diabetes. *ENSA* overexpressed in type 2 diabetes cases while *PMPCA* downregulated in type 2 diabetes cases.
